# Modern modelling techniques are data hungry: a simulation study for predicting dichotomous endpoints

**DOI:** 10.1186/1471-2288-14-137

**Published:** 2014-12-22

**Authors:** Tjeerd van der Ploeg, Peter C Austin, Ewout W Steyerberg

**Affiliations:** Department of Science, Medical Center Alkmaar/Inholland University, Alkmaar, The Netherlands; Institute of Health Policy, Management and Evaluation, University of Toronto, Toronto, Canada; Department of Public Health, Erasmus MC – University Medical Center Rotterdam, Rotterdam, The Netherlands

## Abstract

**Background:**

Modern modelling techniques may potentially provide more accurate predictions of binary outcomes than classical techniques. We aimed to study the predictive performance of different modelling techniques in relation to the effective sample size (“data hungriness”).

**Methods:**

We performed simulation studies based on three clinical cohorts: 1282 patients with head and neck cancer (with 46.9% 5 year survival), 1731 patients with traumatic brain injury (22.3% 6 month mortality) and 3181 patients with minor head injury (7.6% with CT scan abnormalities). We compared three relatively modern modelling techniques: support vector machines (SVM), neural nets (NN), and random forests (RF) and two classical techniques: logistic regression (LR) and classification and regression trees (CART). We created three large artificial databases with 20 fold, 10 fold and 6 fold replication of subjects, where we generated dichotomous outcomes according to different underlying models. We applied each modelling technique to increasingly larger development parts (100 repetitions). The area under the ROC-curve (AUC) indicated the performance of each model in the development part and in an independent validation part. Data hungriness was defined by plateauing of AUC and small optimism (difference between the mean apparent AUC and the mean validated AUC <0.01).

**Results:**

We found that a stable AUC was reached by LR at approximately 20 to 50 events per variable, followed by CART, SVM, NN and RF models. Optimism decreased with increasing sample sizes and the same ranking of techniques. The RF, SVM and NN models showed instability and a high optimism even with >200 events per variable.

**Conclusions:**

Modern modelling techniques such as SVM, NN and RF may need over 10 times as many events per variable to achieve a stable AUC and a small optimism than classical modelling techniques such as LR. This implies that such modern techniques should only be used in medical prediction problems if very large data sets are available.

**Electronic supplementary material:**

The online version of this article (doi:10.1186/1471-2288-14-137) contains supplementary material, which is available to authorized users.

## Background

Prediction of binary outcomes is important in medical research. The interest in the development, validation, and clinical application of clinical prediction models is increasing [1]. Most prediction models are based on logistic regression analysis (LR), but other, more modern techniques, may also be used. Support vector machines (SVM), neural nets (NN) and random forest (RF) have received increasing attention in medical research [2–6], since these hold the promise of better capturing non-linearities and interactions in medical data. The increased flexibility of modern techniques implies that larger sample sizes may be required for reliable estimation. Little is known, however, about the sample size that is needed to generate a prediction model with a modern modelling technique that outperforms more traditional, regression-based modelling techniques in medical data.

Usually, only a relatively limited number of subjects is available for developing prediction models. In 1995, a comparative study on the performance of various prediction models for medical outcomes concluded that the ultimate limitation seemed due to the availability of the information in data. This study used the term “data barrier” [7].

Some researchers aimed to develop a “power law” that can be used to determine the relation between sample size and the discriminatory ability of prediction models in terms of accuracy [8–10]. These studies clarified how a larger sample size leads to a better accuracy. The studies revealed that a satisfactory level of accuracy (the accuracy at infinite sample size +/− 0.01) can be achieved by sample sizes varying from 300 to 16,000 records, depending on the modelling technique and the data structure. The relation between sample size and accuracy was reflected in learning curves. Similarly, the number of events per variable (EPV) has been studied in relation to model performance [11–15].

In the current study, we aimed to define learning curves to reflect the performance of a model in terms of discriminatory ability, which is a key aspect of the performance of prediction models in medicine [16]. We assumed that the discriminatory ability of a model is a monotonically increasing function of the sample size, converging to a maximum at the infinite sample size. We hypothesized that modern, more flexible techniques are more “data hungry” [17] than more conventional modelling techniques, such as regression analysis. The concept of data hungriness refers to the sample size needed for a modelling technique to generate a prediction model with a good predictive accuracy. For fair comparison, we generated reference models with each of the modelling techniques considered in our simulation study.

## Methods

### Patients

We performed a simulation study, based on three patient cohorts.

The first cohort consisted of patients with head and neck cancer who were followed during 15 years for survival (“HNSCC cohort”) [18]. The cohort contained 7 predictor variables (2 dichotomous, 4 categorical and 1 continuous) and a dichotomous (0/1) outcome with an incidence of 601/1282 (46.9%) (Table 1).

**Table 1:** **Cohort characteristics**

	Cohort		
	HNSCC	TBI	CHIP
**Outcome**	5 year survival	6 months mortality	Intracranial findings
**Type**	dichotomous	dichotomous	dichotomous
**Event/total**	601/1282 (46.9%)	386/1731 (22.3%)	243/3181 (7.6%)
**Predictors**	2 dichotomous	4 dichotomous	9 dichotomous
	4 categorial	1 categorial	1 categorial
	1 continuous	4 continuous	2 continuous

The second cohort consisted of patients with traumatic brain injury (“TBI cohort”) [19]. The cohort contained 10 predictor variables (4 dichotomous, 1 categorical and 4 continuous) and a dichotomous outcome with an incidence of 386/1731 (22.3%) (Table 1).

The third cohort consisted of patients suspected of head injury who underwent a CT-scan (“CHIP cohort”) [6]. This cohort contained 12 predictor variables (9 dichotomous, 1 categorical and 2 continuous) and a dichotomous (0/1) outcome with an incidence of 243/3181 (7.6%) (Table 1).

We generated artificial cohorts by replicating the HNSCC cohort 20 times, the TBI cohort 10 times and the CHIP cohort 6 times. This resulted in an artificial cohort consisting of 25,640 subjects (“HNSCC artificial cohort”), an artificial cohort consisting of 17,310 subjects (“TBI artificial cohort”) and an artificial cohort consisting of 19,086 subjects (“CHIP artificial cohort”).

### Reference models

In the current study, we evaluated the following modelling techniques, using default settings as far as possible:

- Logistic regression (LR)
- Classification and regression trees (CART)
- Support vector machines (SVM)
- Neural nets (NN)
- Random forest (RF)

For a description of these modelling techniques, based on the work of various authors [12, 15, 20, 21], we refer to Additional file 1.

As reference points for this evaluation, we first applied each modelling technique to each entire artificial cohort in order to generate an LR model, a CART model, an SVM model, an NN model and an RF model. These models were fitted with optimization according to default settings. Next, we generated probabilities of the outcome for each of these reference models. With these probabilities, we generated a new 0/1 outcome by comparing the generated probabilities of each reference model with a random number from a uniform (0,1) distribution. Using this new 0/1 outcome, we evaluated the five modelling techniques. The R-code for the construction of the reference models is in Additional file 2.

### Development and validation

For each of the five modelling techniques, we randomly divided the artificial cohort into a development set and a validation set for performance assessment. Each set consisted of 50% of the subjects of the artificial cohort.

### Simulation design and analysis

We applied the following steps to each of the three artificial cohorts:

1. Development sets were samples of increasing sizes (varying from 200 to the maximum size of the development set with increment 1000), drawn at random from the non-validation part of the artificial cohort.
2. For each of the five modelling techniques we generated a model for each sample, taking the 0/1 outcome of a specific reference model as outcome. We evaluated the predictions on each sample.
3. For each sample, the predictions of the model were evaluated on the validation set, taking the 0/1 outcome of the same reference model as outcome.

We repeated these steps 100 times for each sample size to achieve sufficient stability. We considered each of the five reference models in turn for a fair comparison of each of the modelling techniques. Evaluation of predictive performance focussed on the discriminatory ability according to the area under the Receiver Operating Characteristic curve (AUC). The AUC was determined using the development set (apparent AUC) and the validation set (validated AUC). We calculated optimism as mean apparent AUC minus mean validated AUC.

We defined the maximally attainable AUC (AUCmax) as the validated AUC-value of a model based on the entire development set (50% of the artificial cohort).

**Figure 1:**
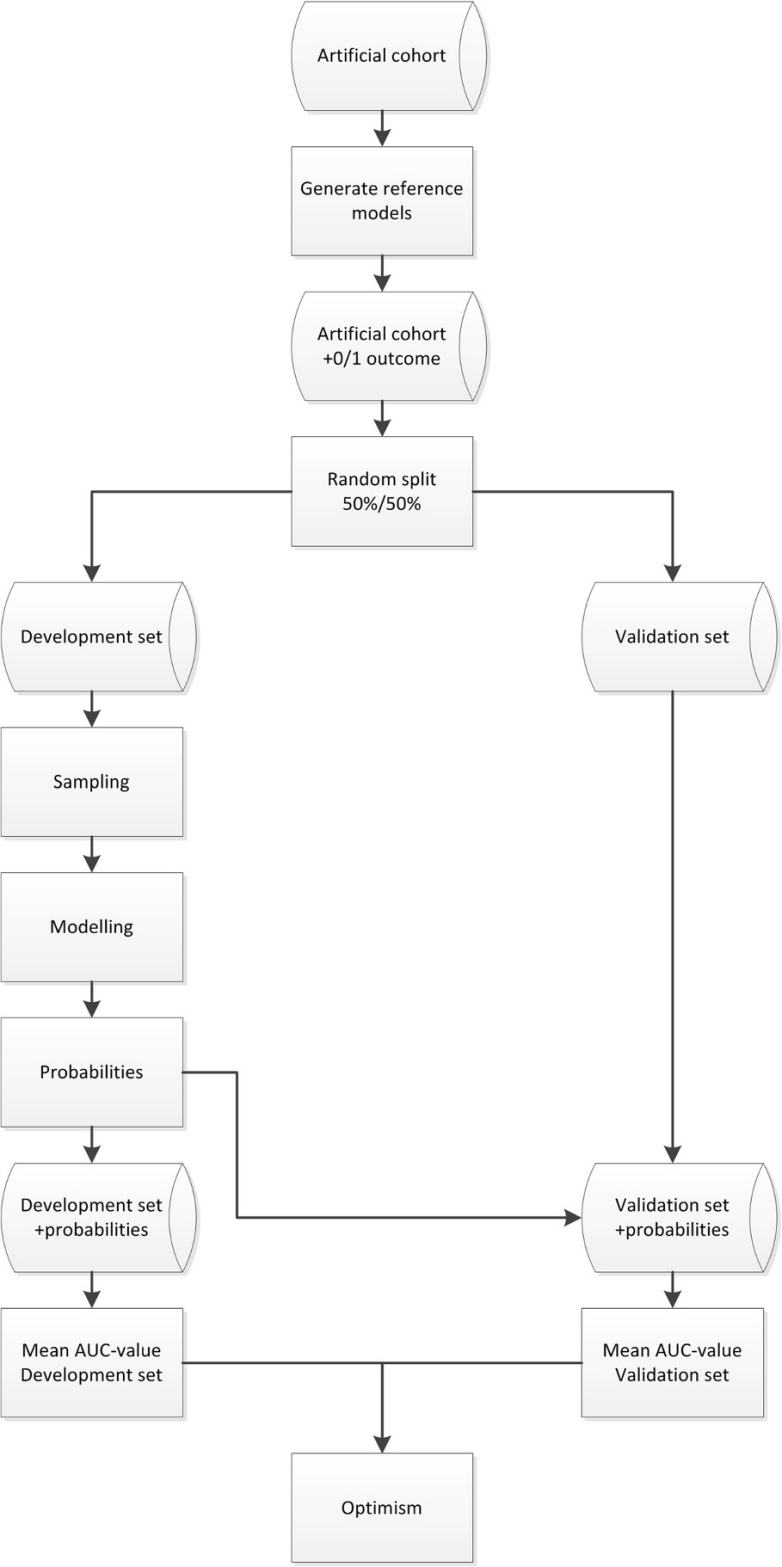
**Flow chart simulation design.**

A flowchart of the simulation design is presented in Figure 1. For the analysis we used R software (version 2.14) [22]. For the R-code of the simulation design we refer to Additional file 2, [23].

### Learning curves

For each modelling technique, we generated learning curves to visualize the relation between the AUC-values and optimism of the generated models with respect to the number of events per variable.

### Data hungriness

The data hungriness of a modelling technique was defined as the minimum number of events per variable at which the optimism of the generated model was <0.01. This limit was admittedly arbitrary, but in line with previous research [24].

### Sensitivity analysis

We performed a sensitivity analysis to determine the influence of the endpoint incidence in the CHIP artificial cohort (7.6%). We hereto selectively oversampled subjects with the outcome of interest in order to generate an artificial cohort with an endpoint incidence of 50% (“CHIP5050 cohort”).

## Results

### HNSCC cohort

The best performance in terms of mean validated AUC-values was achieved when the full development set was used (n = 12,820, number of events = 6013, event rate 46.9%) and by the models generated with the same modelling technique as the reference model, except when the reference model was generated with NN, in which case the RF model had the best performance (AUC 0.810, Table 2).

**Table 2:** **AUCmax per reference model, HNSCC cohort**

	Reference model				
	LR	CART	SV	NN	RF
**LR**	**0.797**	0.745	0.803	0.802	0.880
**CART**	0.730	**0.748**	0.749	0.728	0.822
**SVM**	0.787	0.740	**0.814**	0.802	0.898
**NN**	0.785	0.744	0.800	**0.804**	0.869
**RF**	0.784	0.747	0.810	0.810	**0.929**

Bold numbers are for model performance when the underlying model was specified according to the modelling technique considered.

**Figure 2:**
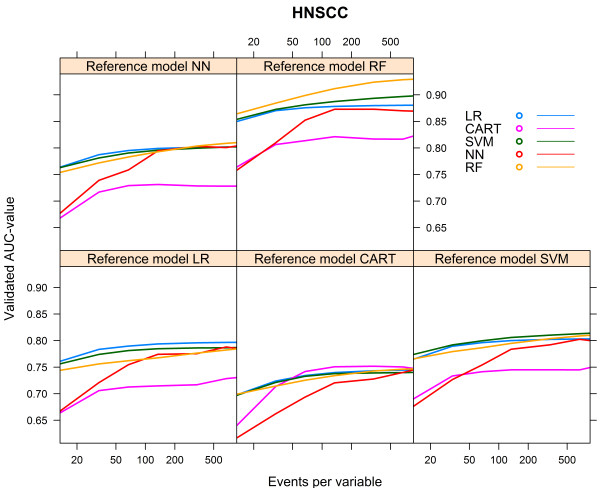
**Validated AUC-values vs. events per variable, HNSCC cohort.**

**Figure 3:**
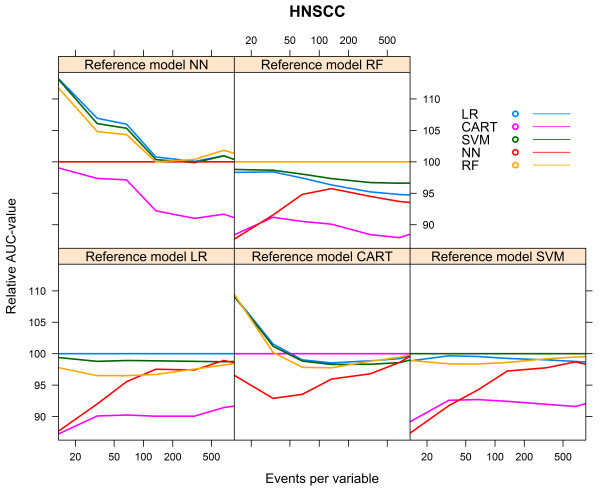
**Relative validated AUC-values vs. events per variable, HNSCC cohort.**

The level that could be reached (AUCmax) depended foremost on the reference model used to generate the 0/1 outcomes. All models performed best when the reference model RF was used. For all reference models, except the CART reference model, the CART model performed worst (Table 2).The data hungriness of the various modelling techniques is reflected by the first part of the learning curves with <100 events per variable (Figure 2). As expected, all models converged monotonically to AUCmax. For each of the reference models, the LR model showed the most rapid increase to a stable mean validated AUC-value, while the RF model needed the largest number of events per variable to reach a stable mean validated AUC-value (Figure 2).We calculated the relative performance of a model by setting the performance of the model resulting from the modelling technique that generated the reference model at 100%. Figure 3 shows the relative performance of the models for each reference model.

For all reference models, the optimism of the models decreased with an increasing number of events per variable. For all reference models, except when the reference model was CART, the modelling technique LR needed the smallest number of events per variable to reach an optimism <0.01 (55 to 127 events per variable).

**Figure 4:**
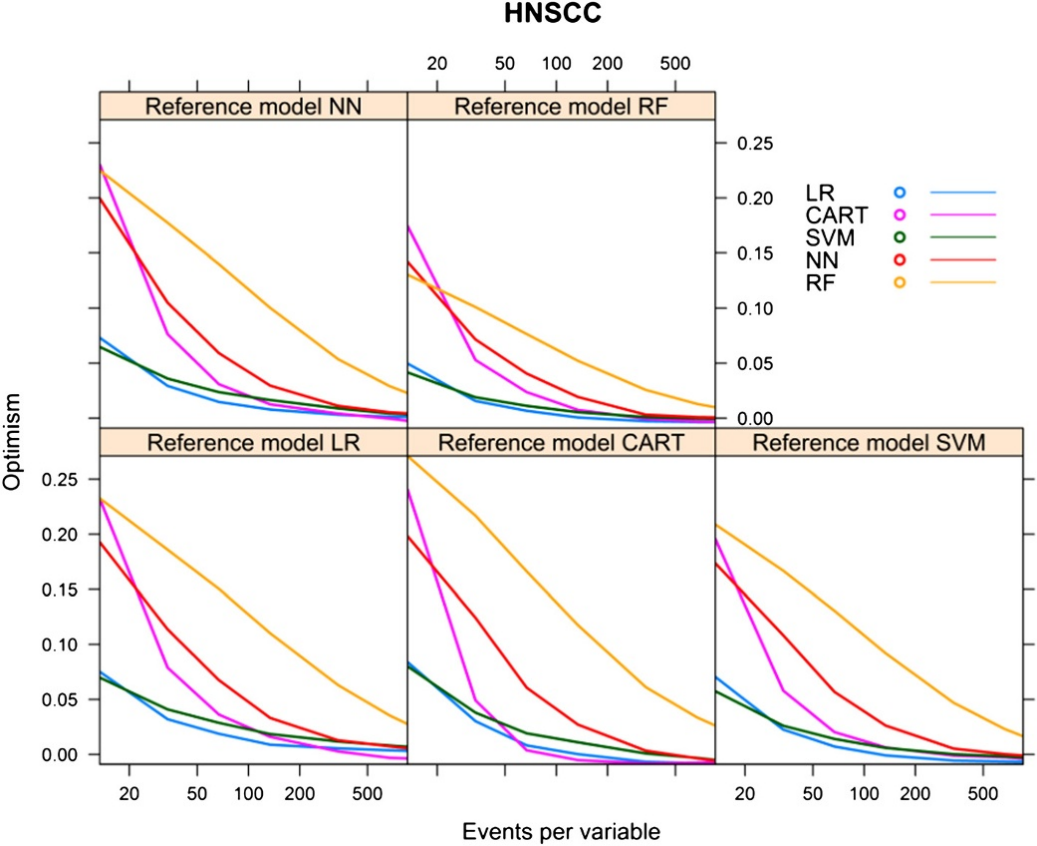
**Optimism vs. events per variable, HNSCC cohort.**

When CART was the reference model, the modelling technique CART needed the smallest number of events per variable to reach an optimism <0.01 (62 events per variable). The modelling techniques NN and RF and, to a lesser extent, SVM needed the most events per variable to generate models with an optimism <0.01.The modelling technique RF needed 850 events per variable when the reference model RF was used, but for the other reference models the optimism of the RF model remained > =0.01, despite the large number of events per variable (Figure 4).

### TBI cohort

**Figure 5:**
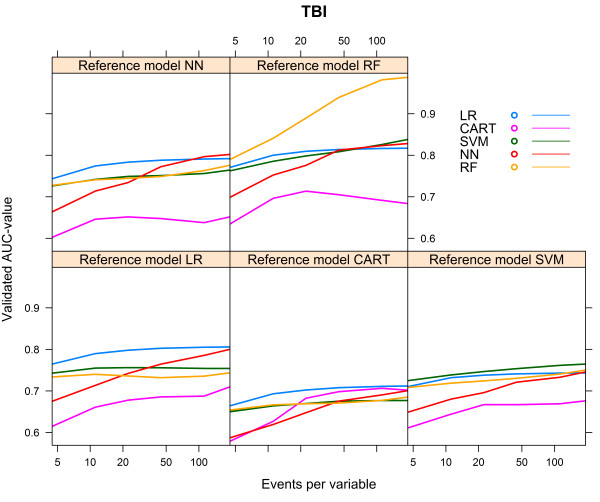
**Validated AUC-values vs. number of events per variable, TBI cohort.**

**Figure 6:**
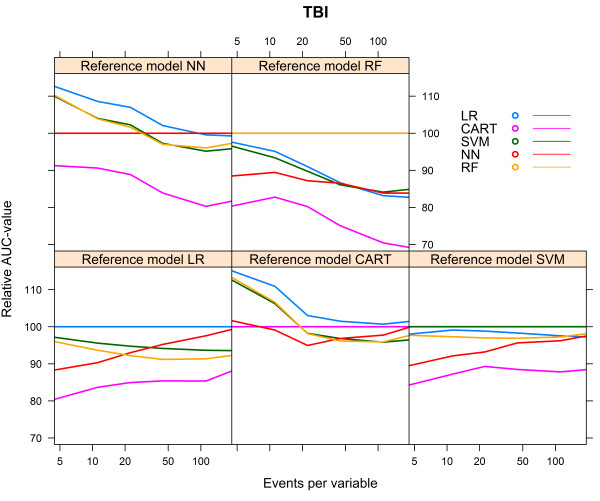
**Relative validated AUC-values vs. events per variable, TBI cohort.**

**Figure 7:**
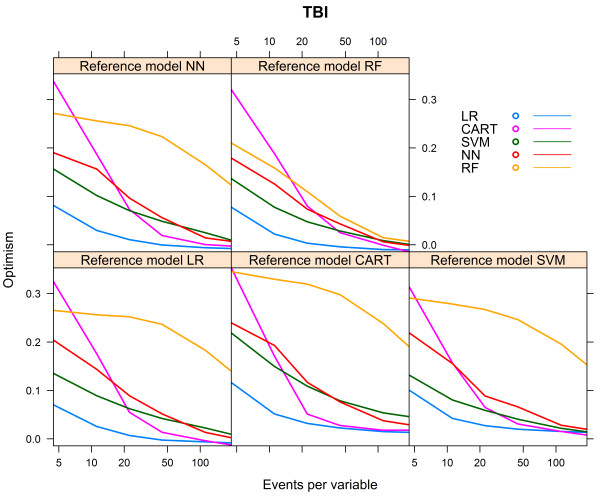
**Optimism vs. events per variable, TBI cohort.**

For the TBI artificial cohort, with a development set consisting of 8655 subjects and 1930 events (event rate 22.3%), the CART models performed poorly, irrespective of the reference model (Table 3). The models generated with the same modelling technique as the reference model showed the best performance, except when the reference model was generated with CART, in which case the LR model had the best performance (AUC 0.712, Table 3). All models, except the CART model, showed the lowest AUC when the reference model CART was used (Table 3).The NN model needed the largest number of events per variable to reach AUCmax. For each of the reference models, the LR model showed the most rapid increase to a stable AUC (Figure 5).Again, we calculated the relative performance of a model by setting the performance of the model resulting from the modelling technique that generated the reference model at 100%. Figure 6 shows the relative performance of the models for each reference model.For all models, optimism decreased with an increasing number of events per variable. The LR model needed 18–23 events per variable to reach an optimism <0.01, whereas the optimism of the RF model remained high, except for the reference model RF, in which case optimism was <0.01 at 163 events per variable (Figure 7).

**Table 3:** **AUCmax per reference model, TBI cohort**

	Reference model				
	LR	CART	SVM	NN	RF
LR	**0.806**	0.712	0.743	0.762	0.817
CART	0.710	**0.702**	0.676	0.652	0.684
SVM	0.754	0.677	**0.765**	.0765	0.838
NN	0.800	0.701	0.746	**0.802**	0.828
RF	0.744	0.685	0.750	0.776	**0.988**

Bold numbers are for model performance when the underlying model was specified according to the modelling technique considered.

### CHIP cohort

**Figure 8:**
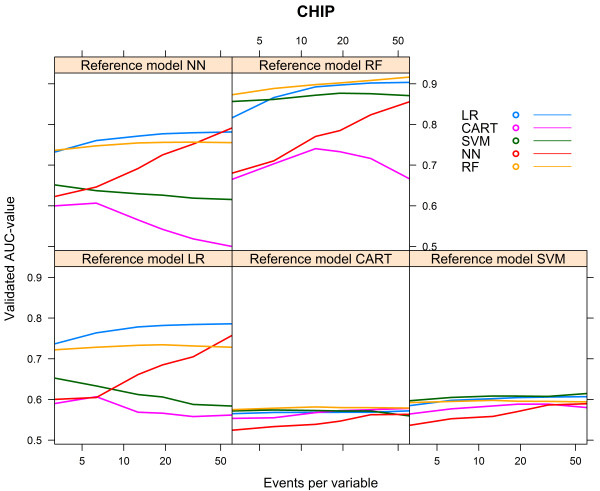
**Validated AUC-values vs. events per variable, CHIP cohort.**

**Figure 9:**
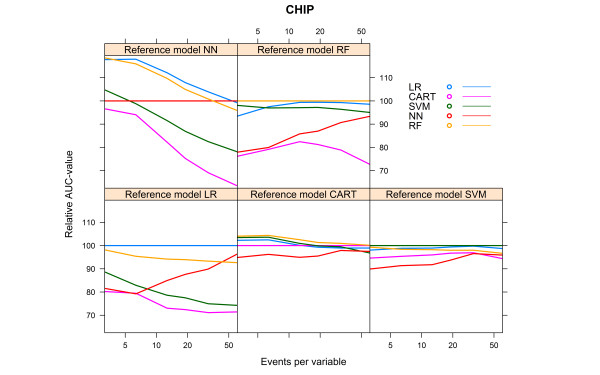
**Relative validated AUC-values vs. events per variable, CHIP cohort.**

**Figure 10:**
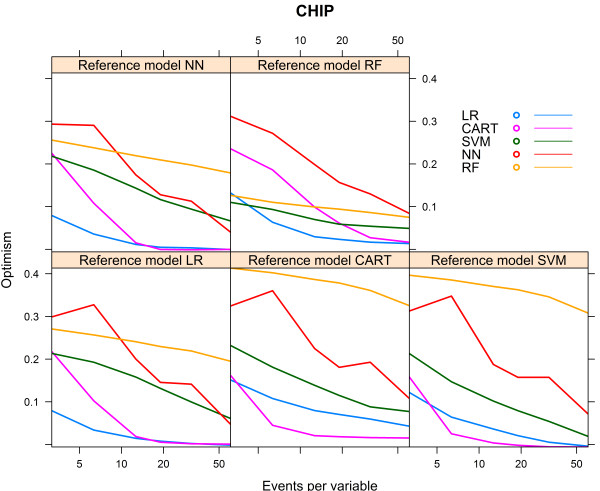
**Optimism vs. events per variable, CHIP cohort.**

For the CHIP artificial cohort, with a development set consisting of 9543 subjects and 729 events (event rate 7.64%), the findings were largely similar to the results of the HNSCC cohort. The best performance was achieved by the same modelling technique that generated the reference model (Table 4). The modelling technique CART generated models with a poor performance, irrespective of the reference models. The modelling technique SVM also generated models with a poor performance, irrespective of the reference models, except when the RF model was used as reference model (AUC 0.871, Table 4). All models performed poorly when the reference models CART and SVM were used. All models, except the CART model, performed well when the reference model RF was used (AUC > 0.8, Table 4).Considering the learning curves (Figure 8), the CART models performed poorly. For each of the reference models, the LR model showed a rapid increase to a stable mean validated AUC-value, in contrast to the NN model which needed far more events to reach a stable mean validated AUC-value. The CART model showed a decreasing mean validated AUC-value despite increasing number of events, except when the reference model CART was used (Figure 8).Figure 9 shows the relative performance of the models for each reference model.For the reference models LR, SVM and NN, the modelling technique LR required 14 to 28 events per variable to reach an optimism <0.01 and CART required 11 to 17 events per variable. Despite an increasing number of events per variable, the modelling techniques SVM, NN and RF generated models with optimism >0.01 for all reference models. For the reference models CART and RF, none of the modelling techniques was able to generate a model with optimism <0.01 (Figure 10).

**Table 4:** **AUCmax per reference model, CHIP cohort**

	Reference model				
	LR	CART	SVM	NN	RF
LR	0.786	0.572	0.607	0.782	0.903
CART	0.562	**0.578**	0.580	0.500	0.666
SVM	0.584	0.560	**0.615**	0.616	0.871
NN	0.758	0.564	0.589	**0.791**	0.856
RF	0.728	0.579	0.594	0.755	**0.916**

Bold numbers are for model performance when the underlying model was specified according to the modelling technique considered.

### Sensitivity analysis CHIP cohort

When we increased the event rate in the CHIP cohort from 7.6% to 50% (“CHIP5050 cohort”), the behaviour of the learning curves became largely similar to the behaviour of the curves generated for the HNSCC cohort (Additional file 3, Figures 11, 12 and 13).

**Figure 11:**
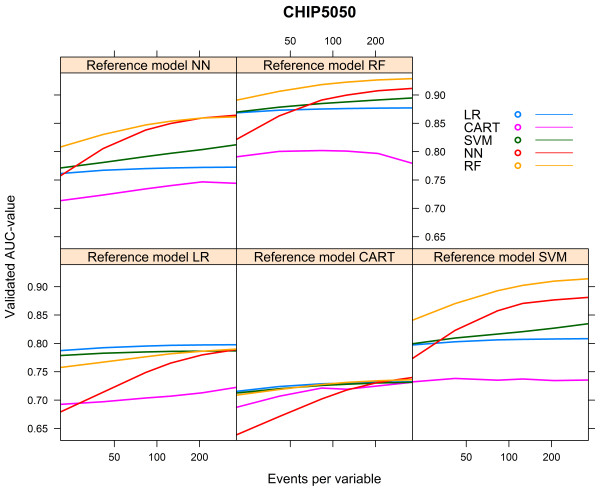
**Validated AUC-values vs. events per variable, CHIP5050 cohort.**

**Figure 12:**
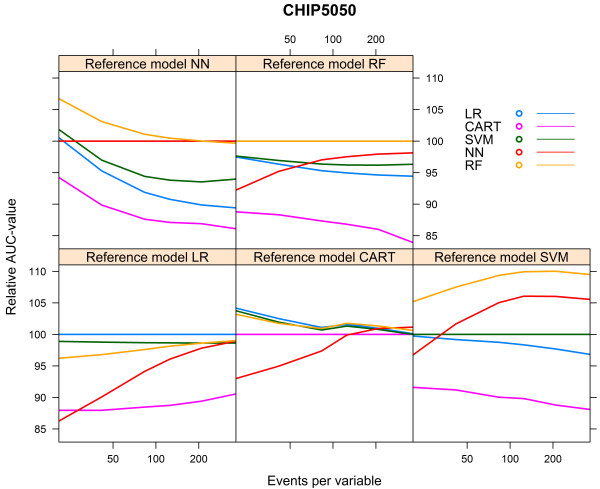
**Relative validated AUC-values vs. events per variable, CHIP5050 cohort.**

**Figure 13:**
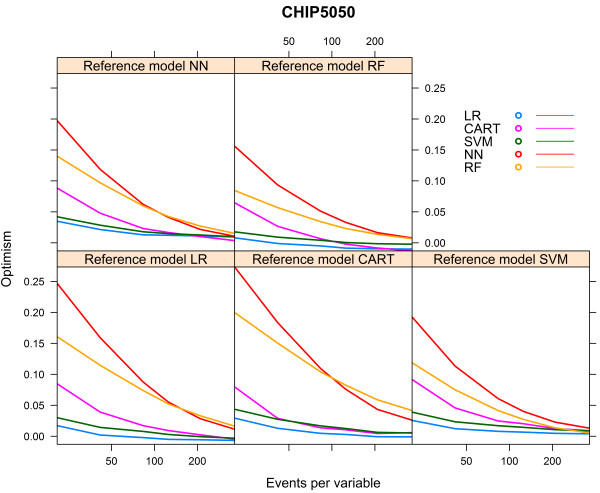
**Optimism vs. events per variable, CHIP5050 cohort.**

## Discussion

Modern modelling techniques, such as SVM, NN and RF, needed far more events per variable to achieve a stable validated AUC and an optimism <0.01 than the more conventional modelling techniques, such as LR and CART. The CART models had a stable performance, but at a fairly poor level. Specifically, a larger number of events did not lead to better validated performance in the cohort with a 7.6% event rate. The LR models had low optimism when the number of events per variable was at least 20 to 50. A remarkable finding was that the optimism of the RF models remained high for the three cohorts, even at a large number (over 200) of events per variable. This indicates that these RF models were far from robust. Of note, the validated performance of RF models was similar to that of LR models. This implies that especially RF models need careful validation to assess predictive performance, since apparent performance may be highly optimistic.

Since LR modelling is far less data hungry than alternative modelling techniques, this technique may especially be useful in relatively small data sets. With very small data sets, any modelling technique will lead to poorly performing models. Our results confirm the generally accepted rule that reasonable predictive modelling requires at least 10 events per variable, even with a robust technique such as LR [11, 12, 15]. We note that larger numbers of events per variable are desirable to achieve better stability and higher expected performance.

The modelling techniques SVM and NN needed far more events per variable to generate models with a stable mean validated AUC-value and an optimism converging towards zero. For models generated with the modelling technique RF, the optimism did not even converge towards zero at the largest number of events per variable that we evaluated.

Obviously, models generated by the same modelling technique as the reference model generally performed best, reflecting a “home advantage” over models generated by a different modelling technique than the reference model. The performance of models according to different reference models was provided for a fair assessment of the performance of the approaches considered.

While RF and LR models consistently performed well, CART consistently performed poorly. The poor performance of CART modelling may be explained by the fact that continuous variables need to be categorized, with optimal cut-offs determined from all possible cut-off points, and that possibly unnecessary higher-order interactions are assumed between all predictor variables. RF modelling is an obvious improvement over CART modelling [24]. It is hence remarkable that CART is still advocated as the preferred modelling technique for prediction in some disease areas, such as trauma [25]. A researcher must always carefully consider which modelling technique is appropriate in a specific situation. Using, for instance, a random forest technique just because the number of subjects is over 10,000 is too simplistic.

The aim of our study was to investigate the data hungriness of the various modelling techniques and the aim was not to find the best modelling technique in AUC terms. To our knowledge, the data hungriness of various modelling techniques has not been assessed before for medical prediction problems. However, a few studies addressed this topic in the context of progressive sampling for the development of a power law to guide the required sample size for prediction modelling. For example, arithmetic sampling was applied with sample sizes of 100, 200, 300, 400 etc. to 11 of the UCI repository databases to obtain insight into the performance of a naive Bayes classifier [8]. This study led to required sample sizes from 300 to 2180 to be within 2% from the accuracy of a model built from the entire database. Other researchers modelled 3 of the larger databases from the UCI repository using different progressive sampling techniques [9]. Using the C4.5 modelling technique, which we consider a CART variant, sample sizes of 2000 for the LED database, 8000 for the CENSUS database and 12000 for the WAVEFORM database were required for a model being no more than 1% less accurate than a model based on all the available data.

Another study compared the performance of 6 data mining tools at various sample sizes for 2 test databases (test database I with 50,000 records and test data base II with 1,500,000 records), using accuracy as the performance measure. For test database I, for all tools, a stable level of accuracy was reached at 16,000 records, and for test database II, for all tools, a stable level was reached at 8000 records [10]. The results of our study are in line with these studies. Although we used mean validated AUC-values instead of accuracy to measure the performance of the models, we also found that the more complex modelling techniques required large numbers of events per variable to generate models with optimism <0.01.

A number of limitations need to be considered. Firstly, we used three cohorts with dichotomous outcomes, in which non-linearity was not a major issue. While this may be common in medical research, it limited the ability for some modern modelling techniques to outperform traditional logistic regression modelling. If important non-linearity is truly present in a data set, techniques that capture such non-linear patterns well are obviously attractive. Various approaches can be considered to address non-linearity within the regression framework, including restricted cubic splines and fractional polynomials [15, 26]. Secondly, we used default settings for the modelling techniques [8]. Further research might investigate our evaluated models, but also other modelling techniques such as LASSO, using other cohorts, and also using other settings for the modelling (such as pruning options, priors, and number of subjects in the end nodes).

Thirdly, there was a considerable difference in incidence between the three cohorts (47%, 22% and 8%). To assess the effect of this difference in incidence on the data hungriness, we performed a sensitivity analysis. Further research should evaluate the relation between the incidence of the outcome and the data hungriness patterns of various modelling techniques.

## Conclusions

Modern modelling techniques such as SVM, NN and RF need far more events per variable to achieve a stable AUC-value than classical modelling techniques such as LR and CART. If very large data sets are available, modern techniques such as RF may potentially achieve an AUC-value that exceeds the AUC-values of modelling techniques such as LR. The improvement over simple LR models may, however, be minor, as was shown in the two empirical examples in this study. This implies that modern modelling techniques should only be considered in medical prediction problems if very large data sets with many events are available.

## Electronic supplementary material

Additional file 1: **Description of the modeling techniques.** (DOCX 30 KB)

Additional file 2: **R-code simulation design and analysis.** (DOCX 29 KB)

Additional file 3: **Results sensitivity analysis.** (DOCX 26 KB)

## Abbreviations

CT: Computed tomography
SVM: Support vector machines
NN: Neural nets
RF: Random forest
CART: Classification and regression trees
LR: Logistic regression
ROC: Receiver operating curve
AUC: Area under the curve
EPV: Events per variable
HNSCC: Head and neck squamous cell carcinoma
TBI: Traumatic brain injury
CHIP: CT in head injury patients
UCI: University of California, Irvine
LASSO: Least absolute shrinkage and selection operator.
