# Supplementary material for: Modern modelling techniques are data hungry: a simulation study for predicting dichotomous endpoints
Source: BMC Med Res Methodol. 2014 Dec 22;14:137. doi: 10.1186/1471-2288-14-137 (PMC4289553; doi:10.1186/1471-2288-14-137)
Supplement: Supplementary file 1 — Additional file 1: Description of the modeling techniques. (DOCX 30 KB) [file 12874_2014_1146_MOESM1_ESM.docx]

**Additional file 1 Description of the modeling techniques**

This appendix describes the evaluated modelling techniques in detail, based on the work of several authors [12] [15] [20] [21].

Logistic regression (LR)

Logistic regression is a type of regression analysis used for predicting the outcome of a binary dependent variable (a variable which can take only two possible outcomes, e.g. "yes" vs. "no" or "success" vs. "failure") based on one or more predictor variables. Logistic regression attempts to model the probability of a "yes/success" outcome using a linear function of the predictors. Specifically, the log-odds of success (the logit of the probability) is fit to the predictors using linear regression. Logistic regression is one type of discrete choice model, which in general predict categorical dependent variables, either binary or multi-way.

Like other forms of regression analysis, logistic regression makes use of one or more predictor variables that may be either continuous or categorical. Also, like other linear regression models, the expected value (average value) of the response variable is fit to the predictors, the expected value of a Bernoulli distribution is simply the probability of success. Unlike ordinary linear regression, however, logistic regression is used for predicting binary outcomes (Bernoulli trials) rather than continuous outcomes, and models a transformation of the expected value as a linear function of the predictors, rather than the expected value itself.

Classification and regression trees (CART)

Classification and regression trees is a tree-based classification and prediction modeling technique which uses recursive partitioning to split the training records into segments with similar output variable values. The modeling starts by examining the input variables to find the best split, measured by the reduction in an impurity index that results from the split. The split defines two subgroups, each of which is subsequently split into two further subgroups and so on, until the stopping criterion is met. The parameter of RPART is the cp-parameter (cost complexity factor). A cp-value of 0.001 for example regulates that a split must decrease the overall lack of fit by a factor of 0.001.

Support vector machine (SVM)

A Support Vector Machine performs classification tasks by constructing hyperplanes with a margin in a multidimensional space that separates cases from different classes. SVM can efficiently perform a non-linear classification or regression task using different kernels (radial, linear and polynomial). The tuning parameters for SVM are the C-parameter (cost), which regulates the margin width, and the gamma-parameter for the kernel calculation. SVM claims to be a robust classification and regression technique that maximizes the predictive accuracy of a model without overfitting the training data. SVM may particularly be suited to analyse data with large numbers of predictor variables.

Neural nets (NN)

A neural network (NN), sometimes called a multilayer perceptron, works by simulating a large number of interconnected simple processing units, which are arranged in layers. There are three parts in a neural network: an input layer, with units representing the predictor variables, one or more hidden layers and an output layer, with a unit representing the outcome variable. The units are connected with varying connection strengths or weights. Input data are presented to the input layer and values are propagated from there to the next layer. Then, a prediction is delivered from the output layer. The network learns by examining individual records, generating a prediction for each record and making adjustments to the weights whenever it makes an incorrect prediction. This process is repeated many times, and the network continues to improve its predictions until one or more of the stopping criteria have been met. Initially, all weights are random, and the predictions that come out of the net are nonsensical. The network learns through training. Records for which the output is known are repeatedly presented to the network, and the predictions it gives are compared to the known outcomes. As training progresses, the network becomes increasingly accurate in replicating the known outcomes. Once trained, the network can be applied to new patients for whom the outcome is unknown. The parameters of NN are the size-parameter (number of units in the layer) and decay-parameter.

Random forest (RF)

Random forest is an ensemble classifier that consists of many decision trees and outputs the class that is the mode of the classes output by individual trees. The algorithm for inducing a random forest was developed by Leo Breiman and Adele Cutler, and "Random Forests" is their trademark.

Each tree is constructed using the following algorithm:

1. Let the number of training cases be N, and the number of variables in the classifier be M.
2. We are told the number m of input variables to be used to determine the decision at a node of the tree; m should be much less than M.
3. Choose a training set for this tree by choosing n times with replacement from all N available training cases (i.e. take a bootstrap sample). Use the rest of the cases to estimate the error of the tree, by predicting their classes.
4. For each node of the tree, randomly choose m variables on which to base the decision at that node. Calculate the best split based on these m variables in the training set.
5. Each tree is fully grown and not pruned (as may be done in constructing a normal tree classifier).

For prediction a new sample is pushed down the tree. It is assigned the label of the training sample in the terminal node it ends up in. This procedure is iterated over all trees in the ensemble, and the average vote of all trees is reported as random forest prediction.
