# Supplementary material for: Modern modelling techniques are data hungry: a simulation study for predicting dichotomous endpoints
Source: BMC Med Res Methodol. 2014 Dec 22;14:137. doi: 10.1186/1471-2288-14-137 (PMC4289553; doi:10.1186/1471-2288-14-137)
Supplement: Supplementary file 2 — Additional file 2: R-code simulation design and analysis. (DOCX 29 KB) [file 12874_2014_1146_MOESM2_ESM.docx]

**Additional file 2 R-code simulation design and analysis**

This appendix describes the R-code that was used for the simulation design on the HNSCC artificial cohort with a LR model as reference.

**# Open libraries**

library(foreign)

library(KsPlot)

**# Cohort creation**

HNSCC=read.spss("HNSCC20x.sav",use.value.labels=FALSE,to.data.frame=TRUE)

Gender=as.factor(HNSCC$Gender)

Tumor_location=as.factor(HNSCC$Tumor_location)

T_class=as.factor(HNSCC$T_class)

N_class=as.factor(HNSCC$N_class)

M_class=as.factor(HNSCC$M_class)

Prior_malignancies=as.factor(HNSCC$Prior_malignancies)

Age_at_diagnosis=as.numeric(HNSCC$Age_at_diagnosis)

ACE27=as.factor(HNSCC$ACE27)

Dead_or_alive_at_60_months=as.numeric(HNSCC$Dead_or_alive_at_60_months)

HNSCC2<-data.frame(Gender,Tumor_location,T_class,N_class, Prior_malignancies,Age_at_diagnosis,ACE27,Dead_or_alive_at_60_months)

**# Construction of a binary outcome with the LR model as reference model**

lrModel <- glm(as.factor(Dead_or_alive_at_60_months)~ ., data = HNSCC2, family = "binomial")

lrProbs <- predict(lrModel, HNSCC2, type = "response")

lrROC <- caTools::colAUC(lrProbs,HNSCC2$Dead_or_alive_at_60_months)

lrROC

set.seed(1)

runis = runif(25640,0,1)

lry = ifelse(runis < lrProbs,1,0)

BASE<-data.frame(lry,Gender,Tumor_location,T_class,N_class, Prior_malignancies,Age_at_diagnosis,ACE27)

**# Creation development set and validation set**

Sample <- sample(1:nrow(BASE), nrow(BASE)/2)

devBASE<- BASE[Sample, ]

valBASE<- BASE[-Sample, ]

**# Modeling with the modeling techniques LR, CART, SVM, NN and RF with increasing sample size**

output <- matrix(NA, nrow = 700, ncol=12, byrow=TRUE, dimnames = list(c(1:700),c("Sample number per size", "Sample size", "lrROCtraining","lrROCtest","cartROCtraining","cartROCtest","svmROCtraining","svmROCtest","nnROCtraining","nnROCtest","rfROCtraining", "rfROCtest")))

k=1

for( j in c(200, 500, 1000, 2000, 5000, 10000,nrow(devBASE)))

{

for (i in 1:100)

{

sampledata=devBASE[sample(1:nrow(devBASE),j),]

lrModel <- glm(as.factor(lry)~ ., data = sampledata, family = "binomial")

lrProbs1 <- predict(lrModel, sampledata, type = "response")

lrProbs2 <- predict(lrModel, valBASE, type = "response")

lrROCtraining<- caTools::colAUC(lrProbs1,sampledata$lry)

lrROCtest <- caTools::colAUC(lrProbs2,valBASE$lry)

cartModel <- mvpart::rpart(as.factor(lry)~., data = sampledata)

cartProbs1 <- predict(cartModel, sampledata)

cartProbs2 <- predict(cartModel, valBASE)

cartROCtraining<- caTools::colAUC(cartProbs1[,2],sampledata$lry)

cartROCtest <- caTools::colAUC(cartProbs2[,2],valBASE$lry)

svmModel <- e1071::svm(lry ~ ., data = sampledata,kernel = "polynomial", degree = 3, probability = T)

svmProbs1 <- predict(svmModel, sampledata, probability = T)

svmProbs2 <- predict(svmModel, valBASE, probability = T)

svmROCtraining<- caTools::colAUC(svmProbs1,sampledata$lry)

svmROCtest <- caTools::colAUC(svmProbs2,valBASE$lry)

nnModel <- nnet::nnet(as.factor(lry) ~ ., data = sampledata, size = 10)

nnProbs1 <- predict(nnModel, sampledata)

nnProbs2 <- predict(nnModel, valBASE)

nnROCtraining<- caTools::colAUC(nnProbs1,sampledata$lry)

nnROCtest <- caTools::colAUC(nnProbs2,valBASE$lry)

rfModel <- randomForest::randomForest(lry ~ ., data = sampledata)

rfProbs1 <- predict(rfModel, sampledata)

rfProbs2 <- predict(rfModel, valBASE)

rfROCtraining<- caTools::colAUC(rfProbs1, sampledata$lry)

rfROCtest <- caTools::colAUC(rfProbs2, valBASE$lry)

output[k,]<-c(i,j,lrROCtraining,lrROCtest,cartROCtraining,cartROCtest,svmROCtraining,svmROCtest,nnROCtraining,nnROCtest,rfROCtraining, rfROCtest)

print(k)

k=k+1

}

}

**# Performance results to output file**

output

write.csv(output, "HNSCC training and test x vs lr.csv")
