# Supplementary material for: Modern modelling techniques are data hungry: a simulation study for predicting dichotomous endpoints
Source: BMC Med Res Methodol. 2014 Dec 22;14:137. doi: 10.1186/1471-2288-14-137 (PMC4289553; doi:10.1186/1471-2288-14-137)
Supplement: Supplementary file 3 — Additional file 3: Results sensitivity analysis. (DOCX 26 KB) [file 12874_2014_1146_MOESM3_ESM.docx]

**APPENDIX 3 Results sensitivity analysis**

This appendix shows the figures resulting from the sensitivity analysis.

<Figure 11 Validated AUC-values vs. events per variable, CHIP5050 cohort>

<Figure 12 Relative validated AUC-values vs. events per variable, CHIP5050 cohort>

<Figure 13 Optimism vs. events per variable, CHIP5050 cohort>
